# Spin-decoupled metasurface for simultaneous detection of spin and orbital angular momenta via momentum transformation

**DOI:** 10.1038/s41377-021-00497-7

**Published:** 2021-03-25

**Authors:** Yinghui Guo, Shicong Zhang, Mingbo Pu, Qiong He, Jinjin Jin, Mingfeng Xu, Yaxin Zhang, Ping Gao, Xiangang Luo

**Affiliations:** 1 State Key Laboratory of Optical Technologies on Nano-Fabrication and Micro-Engineering, Institute of Optics and Electronics, Chinese Academy of Sciences, Chengdu, 610209 China; 2 School of Optoelectronics, University of Chinese Academy of Sciences, Beijing, 100049 China

**Keywords:** Metamaterials, Sub-wavelength optics, Nanophotonics and plasmonics

## Abstract

With inherent orthogonality, both the spin angular momentum (SAM) and orbital angular momentum (OAM) of photons have been utilized to expand the dimensions of quantum information, optical communications, and information processing, wherein simultaneous detection of SAMs and OAMs with a single element and a single-shot measurement is highly anticipated. Here, a single azimuthal-quadratic phase metasurface-based photonic momentum transformation (PMT) is illustrated and utilized for vortex recognition. Since different vortices are converted into focusing patterns with distinct azimuthal coordinates on a transverse plane through PMT, OAMs within a large mode space can be determined through a single-shot measurement. Moreover, spin-controlled dual-functional PMTs are proposed for simultaneous SAM and OAM sorting, which is implemented by a single spin-decoupled metasurface that merges both the geometric phase and dynamic phase. Interestingly, our proposed method can detect vectorial vortices with both phase and polarization singularities, as well as superimposed vortices with a certain interval step. Experimental results obtained at several wavelengths in the visible band exhibit good agreement with the numerical modeling. With the merits of ultracompact device size, simple optical configuration, and prominent vortex recognition ability, our approach may underpin the development of integrated and high-dimensional optical and quantum systems.

## Introduction

From the perspective of quantum mechanics, photons can carry both spin angular momentum (SAM) and orbital angular momentum (OAM). SAM is manifested as a circular polarization state with a bounded value of ±ℏ per photon^1^, whereas OAM is associated with the “twist” of a helical phase front of exp(*ilφ*) and has unbounded states denoted by *l*ℏ (*φ* is the azimuth, *l* is the mode number and ℏ is Planck’s constant divided by 2*π*)^2–4^. With inherent orthogonality, both SAMs and OAMs of light have been utilized to expand the dimensions of quantum information^5^, optical communications^6–8^, and signal processing^9–12^, wherein unambiguous SAM and OAM identification is one of the significant topics.

Compared with the direct generation of OAM modes through various optical elements, such as spiral phase plates^13^, form-birefringent elements^14,15^, computer-generated holograms^16^, spatial light modulators (SLMs), and ultrathin and planar metasurfaces^17–20^, OAM identification is challenging due to the missing phase information for traditional intensity detectors. The earliest OAM diagnosis strategies were proposed based on interference with the mirror image or a reference wavefront^21–24^, diffraction through specific apertures^25–28^, and reciprocal projection through a forked diffraction grating^5^. Various vortices have been successfully detected, including single-photon vortices^21,23^, supercontinuum femtosecond vortices, noninteger vortices^26^, ultrabroadband vortices^28^, and entanglement vortices^5^. However, most of the aforementioned approaches require complicated optical setups or bulky devices. Furthermore, counting the number of interference fringes and multiple repeated projection measurements were inefficient for the detection of various possible OAM modes, especially for high-order OAMs^29^.

Recently, with the appearance of on-chip plasmonic nanostructures^30^ and metasurfaces^17,31^, compact OAM detection strategies have been proposed^18,19,32^, exhibiting additional merits of ease of interfacing with photodiodes^30,33^ and robustness to certain misalignments^34,35^. Multichannel or superimposed OAM recognition^36,37^ and simultaneous detection of SAMs and OAMs have been demonstrated^35,37^. Nevertheless, restricted by the wavevector-matching condition, on-chip plasmonic nanogratings operate only at a specific wavelength. On the other hand, the detectable OAM modes of dielectric metasurfaces are generally no more than ten^37^.

Alternatively, optical transformation systems that translate different OAMs into separated or distinct intensity patterns on a transverse plane have been investigated. Theoretically, the detectable mode space of the optical transformation methodology is not limited, and it has demonstrated mode detection up to 100 orders^38^. Optical transformations generally require two or more optical elements separated by specific distances with precise alignment. Typically, to perform log-polar transformation^39–42^, one element converts helical OAMs into a beam with a transverse phase gradient, and the other element performs a phase correction and completes the transformation. Most recently, excellent advances have been made for alleviating the mode overlap via spiral transformation^43,44^ and for decreasing the number of utilized optical elements via a single angular lens^45^. However, the above optical transformations mainly rely on bulky refractive elements or large diffractive elements and cannot simultaneously distinguish SAM and OAM. Quite recently, metasurface versions of the log-pol and spiral transformation sorters have been presented for total angular momentum sorting at 1310 and 1550 nm^46,47^, respectively.

In this paper, a single azimuthal-quadratic metasurface-based photonic momentum transformation (PMT) principle is illustrated with detailed mathematical deductions and theoretical analyses. Different vortices in a large mode space are converted into focusing patterns with distinct azimuthal coordinates on a transverse plane via PMT, and thus, OAMs can be determined through a single-shot measurment. Moreover, spin-controlled dual-functional PMTs are proposed for simultaneous SAM and OAM sorting, which is implemented by a single spin-decoupled metasurface that merges both the geometric phase and dynamic phase. With the simultaneous SAM and OAM sorting ability, our proposed method can detect cylindrical vortex vector beams (CVVBs) with both phase and polarization singularities, which may find potential applications in singular optics. Furthermore, our methodology can sort superimposed OAM modes with a certain interval step. For proof-of-concept demonstrations, dielectric metasurfaces have been fabricated by high-resolution electron-beam lithography (EBL) and atom-layer deposition (ALD). Experimental results obtained at several wavelengths in the visible band exhibit good agreement with the numerical modeling. Detailed discussions about the operation bandwidth, detection range, and resolving power of our proposed method are also presented.

## Results

### Principle of PMT

A PMT is an optical transformation that maps received OAM modes into rotational focusing patterns on a transverse plane. Through a mathematical deduction (Section 1 of the Supplementary file), we find that the PMT hinges on a single azimuthal-quadratic phase mask (termed the angular lens in ref. ^45^ and implemented by an SLM), which is expressed as:

1 $${\Phi} (\varphi ) = \frac{{l_0}}{2}\varphi ^2$$

where *l*_0_ is the quadratic phase coefficient and *φ* is the azimuthal coordinate defined as tan^−1^(*y*/*x*). For OAM with a topological charge of *l*, the phase accumulated when passing through the phase mask can be written as:

2 $${\Phi} (\varphi ) + l\varphi = \frac{{l_0}}{2}\left( {\varphi + \frac{l}{{l_0}}} \right)^2 - \frac{{l^2}}{{2l_0}} = {\Phi} (\varphi ^{\prime}) - \frac{{l^2}}{{2l_0}}$$

where *φ*′ denotes a new azimuthal coordinate. Neglecting the azimuth-independent term on the right-hand side of the equation, we find that there is only a mode-dependent rotation through the PMT:

3 $$\varphi _l = - \frac{l}{{l_0}}$$

A phenomenological explanation of the azimuthal rotation is illustrated in Section 2 of the Supplementary file. According to Eq. (3), opposite vortices are focused symmetric about azimuthal coordinate *φ* = 0. With an increase in the topologic charge from negative to positive, the focusing pattern rotates clockwise with an azimuthal interval of 1/*l*_0_. If we can record the azimuthal coordinate of the focusing pattern through such a phase mask, then the received OAM mode can be determined as:

4 $$l = - l_0\varphi _l,\varphi _l \in [ - \pi ,\pi ]$$

**Fig. 1:**
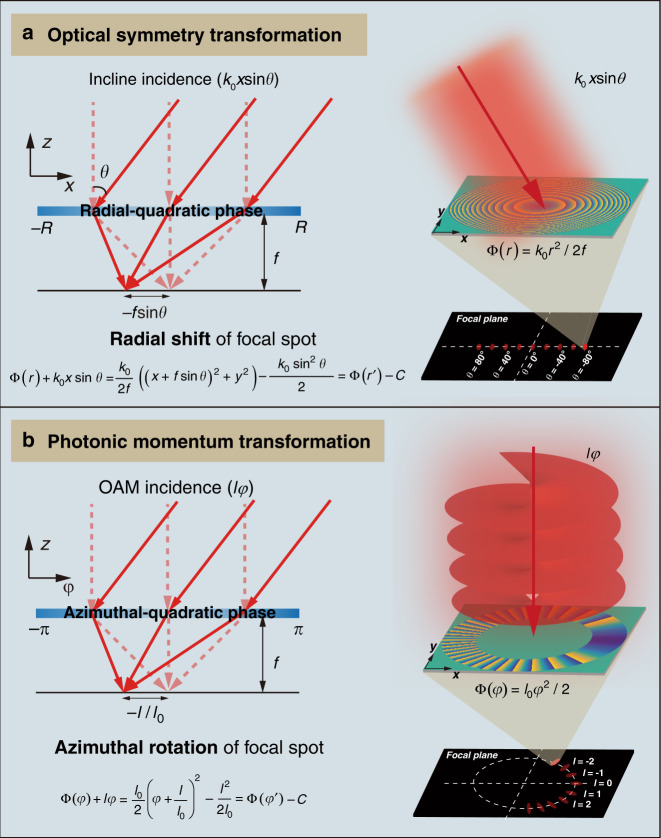
Analogy of two types of optical transformations based on a single quadratic phase mask. **a** Principle of the optical symmetry transformation that translates the rotational symmetry of wide FOV illumination into a transverse symmetry of focus shift, which is utilized for single thin metalens-based wide FOV imaging and wide-angle beam scanning. **b** Principle of the PMT that translates different OAM modes into rotating focusing patterns, which is leveraged for single-metasurface-based single-shot OAM detection

From Eq. (1), it is not easy to deduce the focal distance of the azimuthal-quadratic phase mask. Fortunately, we can obtain it by drawing an analogy between the PMT illustrated here and a similar optical symmetry transformation reported previously^48–50^. As shown in Fig. 1, the optical symmetry transformation is performed through a single radial-quadratic phase mask (${\Phi} \left( r \right) = k_0r^2/2f$), which is mathematically expressed as^48^:

5 $${\Phi} \left( r \right) + k_0x\sin \theta = \frac{{k_0}}{{2f}}\left( {\left( {x + f\sin \theta } \right)^2 \, + \, y^2} \right) - \frac{{k_0\,{\mathrm{sin}}^2\theta }}{2} = {\Phi} \left( {r^\prime } \right) - \frac{{k_0\,{\mathrm{sin}}^2\theta }}{2}$$

where *k*_0_ is the wavevector in free space, *θ* is the oblique angle of the incident light, and *f* is the focal length of the phase mask. The optical symmetry transformation translates off-axis plane waves into focusing spots of different transverse shifts on a focal plane and thus offers a novel design paradigm for single metalens-based extreme field-of-view (FOV) imaging^48,51,52^ and wide-angle beam-steering antennas^49,50^.

With a coordinate transformation (Section 3 of the Supplementary file), the equivalent focal distance of the azimuthal-quadratic phase mask can be determined as:

6 $$f = \frac{{k_0r_0^2}}{{l_0}}$$

where *r*_0_ = (*r*_i_ + *r*_o_)/2 and *r*_*i*_ and *r*_o_ denote the inner and outer radii of the ring phase mask, respectively. According to Eq. (6), the focal length is independent of the topological charge of incident OAM modes, and thus, different OAM modes can be detected on a permanent transverse plane.

### Left-handed circularly polarized (LCP) OAM sorting via a single geometric metasurface

**Fig. 2:**
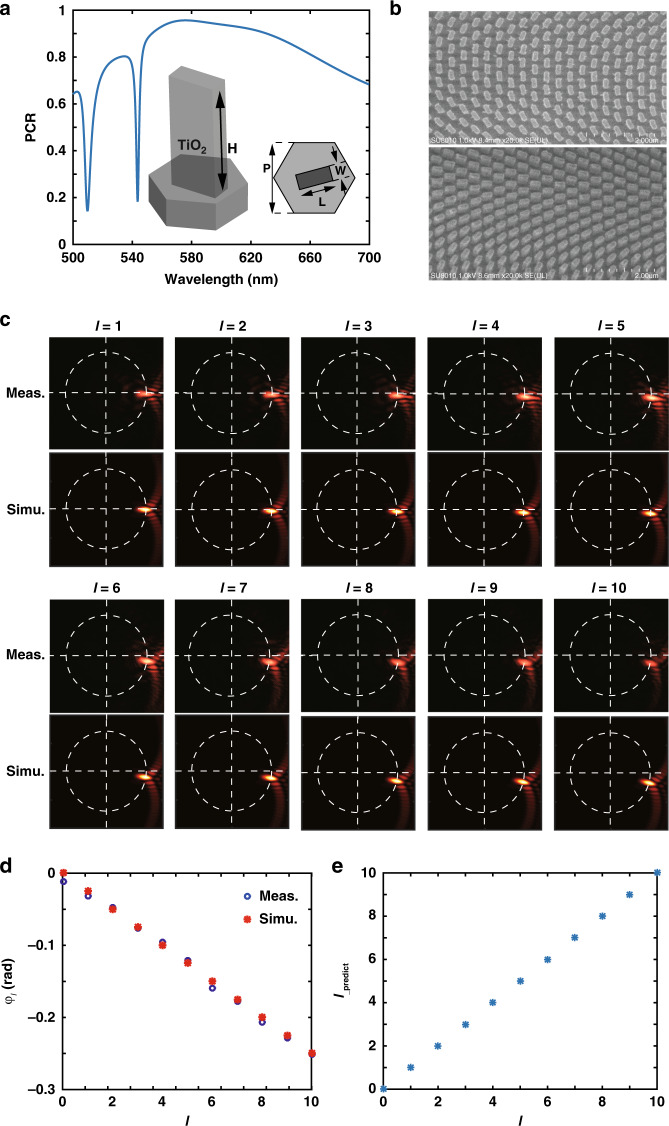
Design and fabrication of geometric metasurfaces and LCP OAM sorting at a wavelength of 633 nm. **a** Simulated polarization conversion ratio (PCR) of the unit cell. Inset: Schematic diagram of the unit cell, which is composed of TiO_2_ nanopillars on a SiO_2_ substrate in a hexagonal lattice. *H* = 800 nm, *P* = 300 nm, *L* = 240 nm, and *W* = 100 nm. **b** Top-view and perspective-view scanning electron microscopy (SEM) images of the fabricated geometric metasurfaces. **c** Measured and simulated focusing patterns for different OAM modes with the topological charge increasing from 1 to 10. **d** Theoretical and measured azimuthal coordinates of the focus peak versus the topological charge. **e** OAM prediction results based on the measured azimuthal coordinates in (**d**)

As illustrated in Fig. 1b, a typical azimuthal-quadratic phase profile changes rapidly toward the azimuthal coordinate of ±*π*, leading to an equivalent wavevector that is several times larger than *k*_0_. Such an azimuthal-quadratic phase mask cannot be easily realized via either refractive or diffractive optics. In contrast to the SLM-based angular lens^45^, a compact geometric metasurface composed of rotating titanium dioxide (TiO_2_) nanopillars in a hexagonal lattice (with C6 symmetry) is leveraged here. The unit cell of the geometric metasurface is shown in the inset of Fig. 2a. TiO_2_ is chosen as the constituent material due to its high refractive index and low loss at visible frequencies. To make the rectangular nanopillars approach an ideal half-wave plate, the geometric parameters of the unit cells are optimized as height *H* = 800 nm, length *L* = 240 nm, width *W* = 100 nm, and period *P* = 300 nm. The pixel size is one order of magnitude smaller than the current commercial SLMs, which helps approach the ideal azimuthal-quadratic phase as much as possible. As indicated in Fig. 2a, the simulated cross-polarization conversion ratio (PCR) is above 90% around 580 nm and gradually decreases away from this wavelength. Therefore, the dispersion of the PCR restricts the operation bandwidth of the geometric metasurface to a certain extent. Nevertheless, the PCR is still >50% in almost the whole visible band.

As an example for proof-of-concept demonstration, a ring-shaped geometric metasurface with *r*_i_ = 50 μm and *r*_o_ = 80 μm (the selection of the radii of the phase mask is discussed in Section 4 of the Supplementary file) is fabricated by etching the complementary patterns into the resist by EBL and conformal growth of high aspect-ratio nanopillars by ALD (see “Metasurface fabrication” in Materials and methods). Top-view and perspective-view scanning electron microscopy (SEM) images of fabricated metasurfaces are shown in Fig. 2b. The in-plane orientation *ξ* of nanopillars for left-handed OAM is determined as:

7 $$\xi = \frac{{{\Phi} \left( \varphi \right)}}{2} = \frac{{l_0\varphi ^2}}{4}$$

Here, *l*_0_ is set to 40 to obtain a large mode recognition space. An OAM sorting experiment is performed for LCP vortices at a wavelength of 633 nm with a continuous-wave He–Ne laser. The geometric phase shift generated by the spin-orbit interaction (SOI) of the geometric metasurface is picked up by the spin-reversed light, and the PMT is performed at a transverse focal plane ~1048.4 µm behind the metasurface, where the focusing pattern is amplified and imaged onto a camera through an objective lens. The experimental setup and measurement procedure are presented in Section 5 of the Supplementary file.

As indicated in Fig. 2c, when the topological charge of the OAM mode increases from 0 to 11, the shape variation of the focusing patterns can be neglected, and there is only a clockwise rotation along the white dotted circle. The results simulated via vectorial angular spectrum theory (see “Numerical modeling” in Materials and methods) are also presented in Fig. 2c and agree well with the measured results. The energy efficiency is limited for several reasons. First, owing to the fabrication defects, the measured diffraction efficiency of the fabricated metasurface is only ~30% (Section 6 of the Supplementary file), which can be further improved by optimizing the fabrication process and adopting semicontinuous metasurfaces^20,53^. Second, since the intensity is spread over a sort of V-shape with a cusp, the amount of energy focused on the specific spot of interest is ~16.3%. Finally, the detection efficiency is fundamentally limited by the intensity fraction in the weak cross-talk area. The detection efficiency of each mode is only ~22.2% (see Section 7 of the Supplementary file). Although it is not high enough for OAM sorting at a single-photon level, it is sufficient for many-photon cases. With careful alignment, the differences between the measured and theoretical azimuthal coordinates are no more than ±0.01 radians (see Section 8 of the Supplementary file). In this situation, the peak intensity is still located within the weak cross-talk region of each mode (the azimuthal separation between the intensity peaks of two adjacent modes is 1/40 = 0.025 radians). Therefore, the topological charge can be unambiguously predicted as:

8 $$l_{{\mathrm{predict}}} = \left[ { - 2l_0\varphi _l} \right]$$

where the operation [•] represents the nearest integer to cancel the deviations caused by the limited pixel size of the camera. The prediction results match the topological charge of the received vortices well, as indicated in Fig. 2e (the influences of misalignment on the detection results are analyzed in Section 9 of the Supplementary file). In addition, simulated results of the PMT for different OAMs with topological charge from 1 to 100 are given in Section 10 of the Supplementary file, demonstrating a large mode detection space of our method.

### Simultaneous SAM and OAM sorting via a single spin-decoupled metasurface

Since the geometric phase is spin-dependent, i.e., the phase profile will be the opposite when the spin is reversed, the geometric metasurface-based PMT only operates under one of the spins. To simultaneously sort SAMs and OAMs, a spin-decoupled dual-functional metasurface that merges dynamic and geometric phases is developed here, which is composed of TiO_2_ rectangular nanopillars with various orientations and geometries. On the one hand, the dynamic phases along the main axes of the rectangular nanopillars can be adjusted by tailoring *L* and *W*. On the other hand, an arbitrary geometric phase can be realized by controlling the local orientation of the fast axes of the nanopillars between 0 and *π*. Owing to the introduction of the spin-independent dynamic phase, the conjugation limitation of the SOI is broken, and thus, dual-functional metasurfaces can be realized^19,54–57^. Distinct from multifunctional metasurfaces constructed by the aperture–division–multiplexing methodology^58,59^, each spin can interact with the whole aperture in spin-decoupled metasurfaces.

**Fig. 3:**
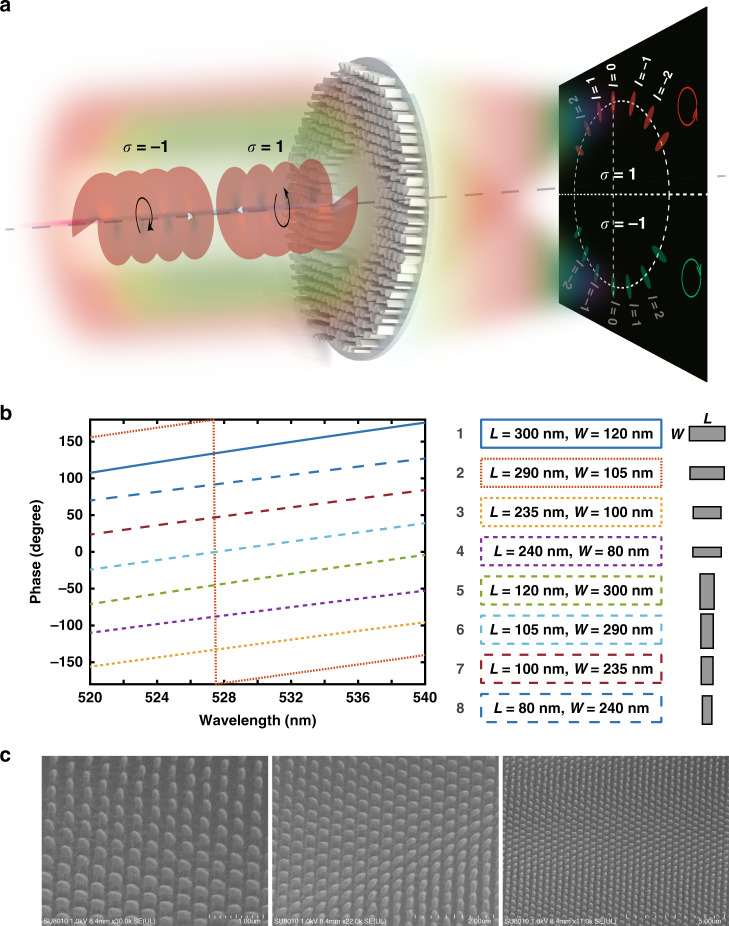
Single spin-decoupled metasurface-based PMTs for simultaneous SAM and OAM sorting. **a** Schematic of a spin-decoupled metasurface that merges the geometric phase and dynamic phase for simultaneous SAM and OAM sorting via spin-controlled PMTs. Vortex beams with different spins are transformed into focusing patterns on two separated halves of the screen on a transverse focal plane with topological charge-dependent azimuthal rotations. **b** Eight unit cells of the spin-decoupled metasurface and their corresponding phase modulation around a wavelength of 532 nm. **c** Perspective-view SEM images of the fabricated spin-decoupled metasurfaces

As shown in Fig. 3a, LCP and right-handed circularly polarized (RCP) incident light picks up spin-decoupled azimuthal-quadric phase profiles as follows:

9a $${\Phi} _{{\mathrm{LCP}}}(\varphi ) = \frac{{l_0}}{2}\left( {\varphi - \frac{\pi }{2}} \right)^2$$

9b $${\Phi} _{{\mathrm{RCP}}}(\varphi ) = \frac{{l_0}}{2}\left( {\varphi + \frac{\pi }{2}} \right)^2$$

and undergoes a pair of center-symmetric PMTs, mapping vortices of different spins into the upper and lower halves of the screen. Therefore, SAMs can be easily sorted by inspecting which half of the screen the focal spot is focused on, and OAMs can be identified by determining the azimuthal coordinate of the focusing patterns, similar to in the demonstration for geometric metasurfaces.

It has been demonstrated by our^19,54,56^ and other groups^57,60^ that to construct spin-decoupled metasurfaces, the anisotropic dynamic phase shifts (*δ*_*x*_ and *δ*_*y*_), and orientation of nanopillars should satisfy:

10a $$\delta _x = \frac{1}{2}\left( {{\Phi} _{{\mathrm{LCP}}} + {\Phi} _{{\mathrm{RCP}}}} \right)$$

10b $$\delta _y = \frac{1}{2}\left( {{\Phi} _{{\mathrm{LCP}}} + {\Phi} _{{\mathrm{RCP}}}} \right) + \pi$$

10c $$\xi = \frac{1}{4}\left( {{\Phi} _{{\mathrm{LCP}}} - {\Phi} _{{\mathrm{RCP}}}} \right)$$

Through parameter sweeping of *L* and *W*, eight nanostructures with the same height *H* = 600 nm and period *P* = 370 nm, including four fundamental nanopillars with different lengths and widths (*L*, *W*) and their orthogonal structures with length and width (*W*, *L*), are selected to provide eight phase levels with an equal phase step of *π*/4 (Fig. 3b) and a high PCR up to 85% around the wavelength of 532 nm (Section 11 of the Supplementary file). SEM images of the fabricated spin-decoupled metasurface are presented in Fig. 3c.

**Fig. 4:**
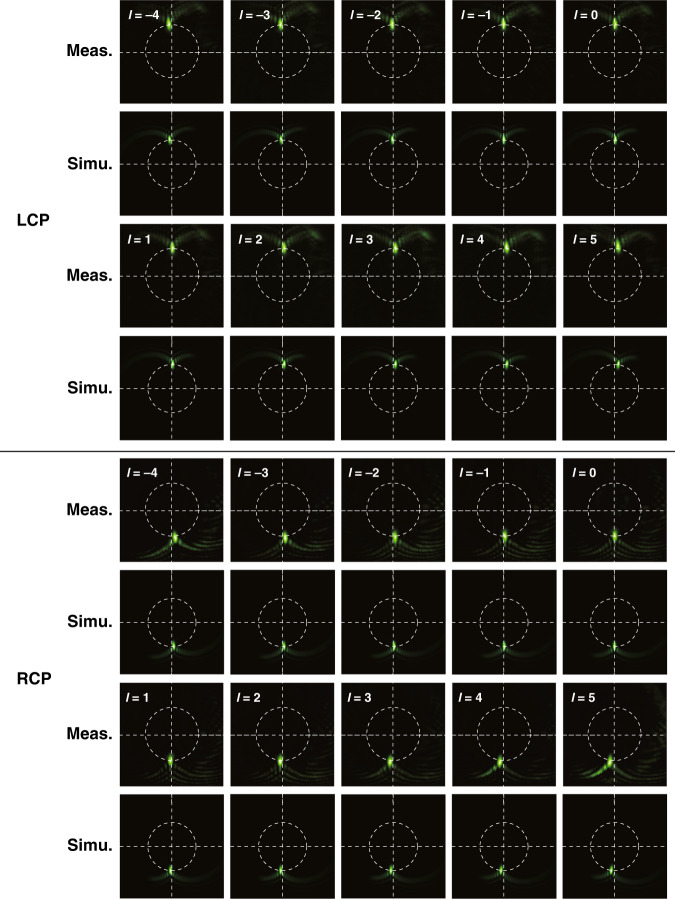
SAM and OAM sorting at a wavelength of 532 nm. Measured and simulated focusing patterns for different SAMs and OAMs with the topological charge changing from −4 to 5

Simultaneous SAM and OAM sorting was performed at a wavelength of 532 nm with a continuous-wave diode-pumped solid-state laser. From Fig. 4, we can see that vortices with different SAMs are mapped into focusing patterns on different halves of the screen, i.e., LCP (*σ* = 1) vortices in the upper half of the screen and RCP (*σ* = −1) vortices in the lower half of the screen; thus, the SAMs can be easily determined. Specifically, for LCP and RCP plane waves (*l* = 0), the focusing patterns are located at *φ* = *π*/2 and *φ* = −*π*/2, respectively. Since the focusing patterns rotate clockwise with increasing topological charge, the OAMs are identified by inspecting the rotation angle relative to the referenced plane wave. The simulated results are also presented in Fig. 4, which are consistent with the measured results.

Intuitively, for linearly polarized vortices that can be taken as superimpositions of LCP vortices and RCP vortices with the same topological charge, there will be two focusing patterns located at two center-symmetric positions on the white dotted circle via spin-decoupled PMTs, which has been demonstrated by the measured results shown in Section 12 of the Supplementary file. A similar sorting experiment was reported in ref. ^61^. The slightly unequal intensities of the two focusing patterns may be attributed to the imperfect fabrication and measurement.

### Sorting of CVVBs with both phase and polarization singularities

**Fig. 5:**
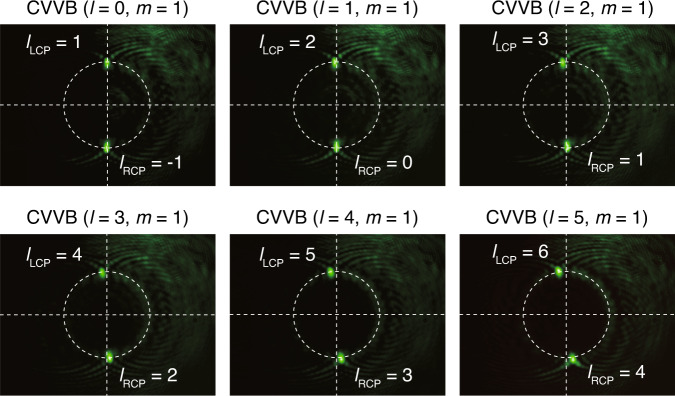
Cylindrical vortex vector beam sorting at a wavelength of 532 nm. Measured focusing patterns for different phase singularities changing from 0 to 5 and polarization singularity *m* = 1

As a family of solutions of the more general vector wave equation^62^, CVVBs feature an inhomogeneous polarization in the dimension transverse to the propagation direction and find important applications in high-resolution imaging and nanoparticle manipulation due to the extra degree of freedom for light manipulation^63^. Theoretically, spin-decoupled metasurface-based dual-functional PMTs can also be utilized to identify CVVBs with phase and polarization singularities, which can be treated as a sum of an LCP vortex with topological charge (*l* + *m*) and an RCP vortex with topological charge (*l* – *m*) according to the Jones matrix of a CVVB^35,64^:

11 $$J_{l,m} = e^{il\varphi }\left( {\begin{array}{*{20}{c}} {\cos \left( {m\varphi + \varphi _0} \right)} \\ {\sin \left( {m\varphi + \varphi _0} \right)} \end{array}} \right) = \frac{1}{2}e^{i\left( {\left( {l + m} \right)\varphi + \varphi _0} \right)}\left( {\begin{array}{*{20}{c}} 1 \\ { - i} \end{array}} \right) + \frac{1}{2}e^{i\left( {\left( {l - m} \right)\varphi + \varphi _0} \right)}\left( {\begin{array}{*{20}{c}} 1 \\ i \end{array}} \right)$$

where *φ*_0_ is the initial phase, *l* is the topological charge, and *m* is the polarization order. To generate the CVVB, we added a vortex wave plate with *m* = 1 (Thorlabs WPV10–532) after the SLM. The measured results in Fig. 5 show that the two focusing patterns in the two halves of the screen are no longer center-symmetric due to the unequal topological charges of the LCP and RCP vortices, demonstrating that full detection of the phase and polarization singularities of the incident beam is achieved. Note that when *l* = 0, the CVVB degenerates into an ordinary cylindrical vector beam (CVB) with only polarization singularity. In this situation, the topological charge values of the LCP and RCP vortices are the same, and the focusing spots are center-symmetric again, which is similar to the observations reported in ref. ^65^.

## Discussion

In subsequent discussions, we take the geometric metasurface as an example, and the conclusions can be easily extended to the spin-decoupled metasurface.

### Sorting of superimposed vortices

Thus far, we have demonstrated a resolving power of Δ*l* = 1 when there is only a single isolated OAM mode received at a time, i.e., there is no cross-talk between adjacent modes. However, when multiple superimposed vortices are simultaneously received, the intensity overlap between them causes a deteriorated resolving power. The simulated and measured results presented in Section 13 of the Supplementary file (Fig. S17) indicate that one cannot resolve two superimposed vortices with a mode interval smaller than Δ*l* = 5, which means that at most 41 superimposed vortices can be simultaneously detected (Fig. S18). Similar mode overlap and resolution limitations were also observed in the log-polar transformation^39^ and angular lens-based OAM sorting methods^45^.

To alleviate mode overlap and improve the sorting resolution, a modified azimuthal-quadratic phase mask is utilized, which is expressed as:

12 $${\Phi}^ \prime (\varphi ) = \frac{{l_0(\varphi /n)^2}}{2}$$

Then, the PMT in Eq. (2) can be rewritten as:

13 $${\Phi}^ {\prime}(\varphi ) + l\varphi = \frac{{l_0}}{{2n^2}}\left( {\varphi + \frac{{n^2l}}{{l_0}}} \right)^2 - \frac{{l^2}}{{2n^4l_0}} = {\Phi}^ {\prime}(\varphi^ {\prime}) - \frac{{l^2}}{{2n^4l_0}}$$

The azimuth of the focusing patterns can be calculated as:

14 $$\varphi _l^\prime = - \frac{{n^2l}}{{l_0}}$$

Equation (14) indicates that the azimuth difference of adjacent OAM modes becomes *n*^2^ times the original one.

By adjusting *n*, an equivalent azimuthal-quadratic phase coefficient *l*_0_/*n*^2^ is realized. According to Eq. (13), the detectable mode range is proportional to *l*_0_/*n*^2^, while the resolving power is inversely proportional to *l*_0_/*n*^2^. With *l*_0_/*n*^2^ decreasing, the mode resolving power for superimposed vortices increases but at the sacrifice of decreasing the detectable mode range. As an example, for a modified phase mask with *n* = 3 (Fig. S19A), the resolving power increases to Δ*l* = 2, while the detection range shrinks to within ±6 orders (Fig. S19B, C). Therefore, the coefficients *l*_0_ and *n* should be selected according to the requirements of the detection range and resolution in practical applications. Details are presented in Section 13 of the Supplementary file. In practical applications, we can realize a mode resolving power of Δ*l* = 1 for a modified phase mask with *n* = 3 by coding the adjacent modes into opposite spins (e.g., $\left| {\sigma _ + } \right\rangle \left| { - 2} \right\rangle$, $\left| {\sigma _ - } \right\rangle \left| { - 1} \right\rangle$, $\left| {\sigma _ + } \right\rangle \left| 0 \right\rangle$, $\left| {\sigma _ - } \right\rangle \left| { + 1} \right\rangle$, $\left| {\sigma _ + } \right\rangle \left| { + 2} \right\rangle$). In addition, our results reported here may have many important applications in not only the measurement of both the spin and angular momentum but also the detection of both phase and polarization singularities. In these scenarios, an isolated vortex mode is generally investigated.

### Broadband PMTs and vortex sorting

A supercontinuum laser source (NKT Photonics, EXP-15) attached to an acoustic optical tuning filter was used to generate light beams across the visible band. Owing to the limited operation band of the SLMs (PLUTO-VIS-014, 420–650 nm) and camera (AmScope MU300, 380–650 nm), the PMTs were experimentally performed at different wavelengths in a range of 480–633 nm. The measured results (Section 14 of the Supplementary file) indicate that both the geometric metasurface and spin-decoupled metasurface can perform mode sorting at these wavelengths, although the spin-decoupled metasurface is optimized at a wavelength of 532 nm.

In addition, we have simulated the longitudinal propagation properties at five wavelengths of 480, 532, 580, 633, and 780 nm. As indicated in Fig. S23A–E, although the focal distances are wavelength-dependent, the depths of focus are sufficiently long such that we can select a transverse plane across all the focal planes. The simulated focusing patterns of 26 superimposed vortices at five different wavelengths on the transverse plane are combined and shown in Fig. S23F, and the corresponding intensity curves along the white dotted line are displayed in Fig. S23G, from which we can see that the peak and valley positions of the focusing patterns at different wavelengths well match each other. These results demonstrate that it is possible to simultaneously detect broadband vortices covering the whole visible band, although additional optical spectral filters are needed for further wavelength identification.

In summary, we have theoretically and experimentally demonstrated a single geometric TiO_2_ metasurface-based OAM mode discrimination method via PMT of the azimuthal-quadratic phase metasurface. Then, we showed that a single spin-decoupled TiO_2_ metasurface that merges the geometric phase and dynamic phase could perform simultaneous SAM and OAM mode discrimination via spin-dependent PMTs, where vortex beams of different spins were transformed into focusing patterns on two separated halves of the screen on a transverse focal plane with topological charge-dependent azimuthal rotations. Further experimental investigations have proven that the single spin-decoupled metasurface possesses the ability to detect cylindrical vortex vector beams with simultaneous phase and polarization singularities. Spin-decoupled PMTs were experimentally demonstrated at several different wavelengths in the visible band. Finally, we showed that the proposed approach could be extended to sorting of superimposed OAMs with a proper mode interval. These results reported here may have many important applications in momentum measurement of both the spin and angular momentum and singularity detection of both phase and polarization singularities.

## Materials and methods

### Numerical modeling

The transversely simulated area is set as 800 × 800 μm^2^, and the grid number *N* in a transverse plane is set as 1024 × 1024. For an arbitrary point (*x*, *y*) in Cartesian coordinates, the corresponding polar coordinates can be expressed as:

15 $$\begin{array}{l}r = \left( {x^2 + y^2} \right)^{1/2}\\ \varphi = \tan ^{ - 1}\left( {y/x} \right)\end{array}$$

An amplitude and azimuthal-quadratic phase mask is utilized:

16 $$M(r,\varphi ) = g\left( r \right)\exp \left( {l_0\varphi ^2/2} \right)$$

where:

17 $$\left\{ {\begin{array}{*{20}{c}} {g\left( r \right) = 1 \quad r_i \le r \le r_o} \\ \!\!\!{g\left( r \right) = 0 \quad {\mathrm{otherwise}}} \end{array}} \right.$$

When a light beam carrying an OAM mode is normally projected on the amplitude and phase mask, the output electric field can be expressed as:

18 $$E_{{\mathrm{out}}}(r,\varphi ) = g\left( r \right)\exp \left( {l_0\varphi ^2/2 + l\varphi } \right)$$

Then, the electric field at an arbitrary transverse plane behind the metasurface can be calculated via vectorial angular spectrum theory^66^:

19 $$E(r_ \bot ,z) = {\int} {{\mathop{\rm{d}}\nolimits} ^2} k_ \bot e^{ik_ \bot \cdot r_ \bot + ik_zz}\left\{ {A_ \bot (k_ \bot ) - [k_ \bot \cdot A_ \bot (k_ \bot )/k_z]e_z} \right\}$$

where $$r_ \bot = xe_x + ye_y, \,$$$$k_ \bot = k_xe_x + k_ye_y, \,$$$$A_ \bot (k_ \bot ) = A_x(k_ \bot )e_x + A_y(k_ \bot )e_y, \,$$$$k_z = \sqrt {k_0^2 - k_x^2 - k_y^2} ,$$ and $$A_ \bot (k_ \bot ) = [{\int} {{\mathop{\rm{d}}\nolimits} ^2} r_ \bot e^{{\mathrm{ - }}ik_ \bot \cdot r_ \bot }E_{{\mathrm{out}}}(r,\varphi )]/(2\pi )^2$$.

### Metasurface fabrication

The fabrication process began with spin-coating a layer of 800-nm-thick electron-beam (e-beam) resist (AR-P 6200) and e-beam evaporation of 10-nm-thick chromium (Cr) onto 540-μm-thick fused silica substrates. Afterward, e-beam lithography was performed at an accelerating voltage of 125 kV and a beam current of 2 nA. The Cr layer was removed by the chromium removal solution, and the samples were developed in o-xylene for 90 s. Atomic layer deposition (ALD) at a low temperature of 100 °C was subsequently used to deposit TiO_2_ onto the developed resist. The overcoated TiO_2_ layer was then etched by reactive ion etching (RIE) with a gas mixture of SF_6_ (20 sccm) and CHF_3_ (5 sccm). Finally, the resist was removed by RIE with O_2_.

## Supplementary information

Supplementary
