# Supplementary material for: Spin-decoupled metasurface for simultaneous detection of spin and orbital angular momenta via momentum transformation
Source: Light Sci Appl. 2021 Mar 25;10:63. doi: 10.1038/s41377-021-00497-7 (PMC7994415; doi:10.1038/s41377-021-00497-7)
Supplement: Supplementary file 1 — Supplementary [file 41377_2021_497_MOESM1_ESM.pdf]

Supplementary Information for

Spin-decoupled metasurface for simultaneous detection of spin and orbital angular momenta via momentum transformation

Yinghui Guo[1,2], Shicong Zhang[1,2], Mingbo Pu[1,2], Qiong He[1], Jinjin Jin[1], Mingfeng Xu[1],Yaxin Zhang[1,2], Ping Gao[1], Xiangang Luo[1,2*]

[1]State Key Laboratory of Optical Technologies on Nano-Fabrication and Micro-Engineering, Institute of Optics and Electronics Chinese Academy of Sciences, Chengdu 610209, China
[2]School of Optoelectronics, University of Chinese Academy of Sciences, Beijing 100049, China
[*]Corresponding author: lxg@ioe.ac.cn

Section 1: A mathematical deduction of the PMT
Section 2: A phenomenological explanation of the PMT
Section 3: A mathematical deduction of the focal distance of the phase mask
Section 4: Discussions on radius choice of the phase mask
Section 5: Experiment setup and measurement procedure
Section 6: Diffraction efficiency of the fabricated geometric metasurface
Section 7: Detection efficiency of the geometric metasurface
Section 8: Measured errors of azimuthal coordinates in Fig. 2(D)
Section 9: Discussions on the centre misalignment in measurements
Section 10: Simulated PMTs of LCP vortices up to 100 order at 633 nm
Section 11: Simulated polarization conversion ratio (PCR) of the spin-coupled metasurface
Section 12: Experimental demonstration of linearly polarized (LP) vortex sorting at 532 nm
Section 13: Sorting of superimposed OAM modes
Section 14: Broadband PMTs and broadband vortex sorting

Section 1: A mathematical deduction of the PMT

The PMT that transforms vortex beams with different OAM modes into in-plane rotation of focusing patterns can be mathematically expressed as:

$$\Phi(\varphi - \Delta\varphi) = \Phi(\varphi) + l\varphi \quad (1)$$

where φ is the azimuthal angle, l is a topological charge of an OAM mode, $\Phi(\varphi)$ represents the phase profile of a mask, and $\Delta\varphi$ is the rotation angle. After simple mathematical transformation, the above equation can be rewritten as:

$$\frac{\Phi(\varphi - \Delta\varphi) - \Phi(\varphi)}{\Delta\varphi} = \frac{l\varphi}{\Delta\varphi} \quad (2)$$

When the $\Delta\varphi$ is infinitely small, the difference operation can be approximately taken as differential operation, i.e.,

$$\frac{\partial\Phi(\varphi)}{\partial\varphi} = -\frac{l}{\Delta\varphi}\varphi \quad (3)$$

Since the rotation angle $\Delta\varphi$ is topological dependent, here we assume:

$$l_0 = -\frac{l}{\Delta\varphi} \quad (4)$$

Then, Eq. (3) can be rewritten as:

$$\frac{\partial\Phi(\varphi)}{\partial\varphi} = l_0\varphi \quad (5)$$

By making a simple integral operation, the phase profile for PMT can be deduced as:

$$\Phi(\varphi) = \frac{l_0\varphi^2}{2} \quad (6)$$

Section 2: A phenomenological explanation of the PMT

A typical azimuthal-quadratic phase profile is shown in Fig. S1(A). It is symmetric about $\varphi_l = 0$ and changes rapidly with the azimuthal coordinates especially approaching ±π, where the equivalent wavevector becomes evanescent. By flattening the azimuthal-quadratic phase profile into a cylindrical-quadratic phase profile, one can easily grasp that such a phase profile will generate a focusing pattern at the azimuthal angle of $\varphi_l = 0$ for a normal plane wave (i.e., $l = 0$). As indicated in Fig. S1(B) and (C), when a light beam carrying an arbitrary OAM mode is normally projected on the phase mask, the symmetric direction of the total phase profile will rotate clockwise or anti-clockwise depending on the incident OAM modes. As a consequence, the propagation wave region and evanescent wave region will interchange, causing a rotation of the focusing pattern.

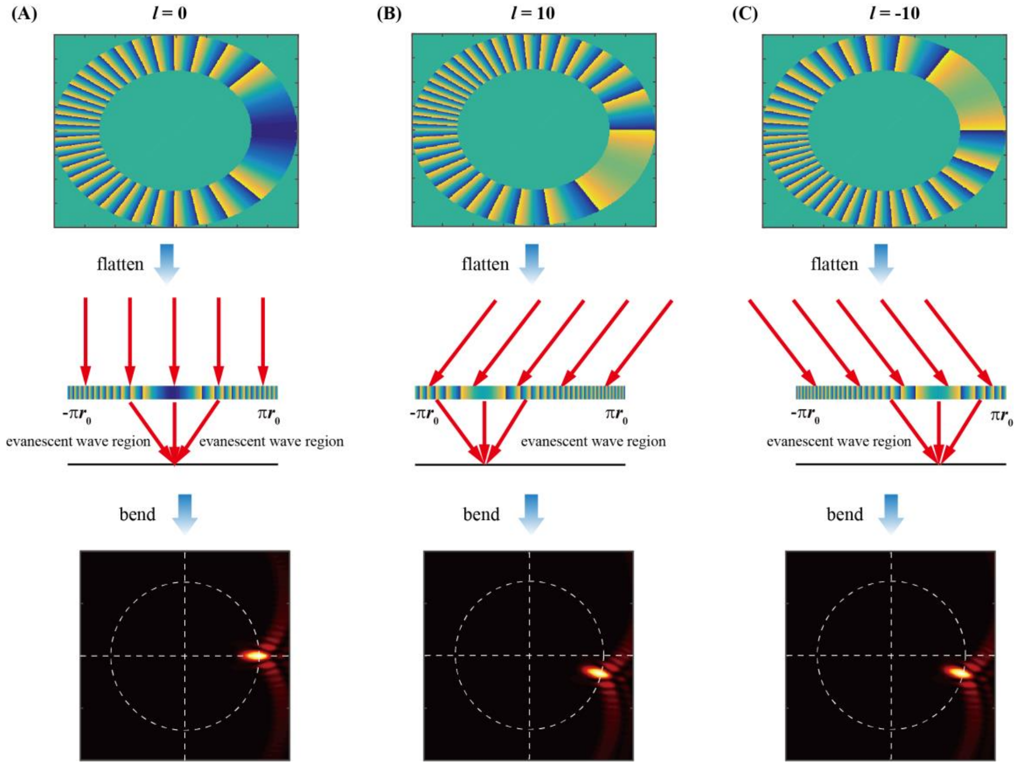

Fig. S1. The phenomenological explanation of PMT. (A)~(C) Accumulated phase profiles after different OAM modes transmitting through the phase mask, and corresponding focusing behaviors in flattening cross plane. (A) $l = 0$, (B) $l = 10$, and (C) $l = -10$.

Section 3: A mathematical deduction of the focal distance of the phase mask

As indicated in Fig. S2, by flattening the Polar coordinates into Cartesian coordinates, we can translate an azimuthal-quadratic phase profile into a cylindrical-quadratic phase profile:

$$\Phi(r) = \frac{l_0}{2}\left(\frac{r}{r_0}\right)^2 \tag{7}$$

Then, the focal distance can be easily determined by drawing an analogy between the flattened phase profile and the phase profile of a cylindrical Fourier lens, expressed as:

$$\frac{l_0}{2}\left(\frac{r}{r_0}\right)^2 = \frac{k_o}{2}\frac{r^2}{f} \tag{8}$$

With a simple mathematical transformation, the focal distance can be deduced as:

$$f = \frac{k_0 r_0^2}{l_0} \tag{9}$$

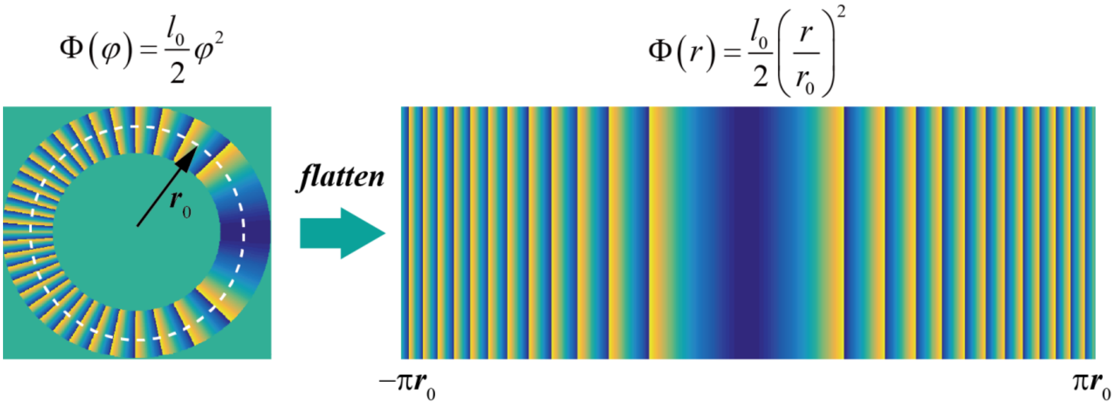

Fig. S2. Flatten the Polar coordinates into Cartesian coordinates. (Left panel) azimuthal-quadratic phase profile and (Right panel) after flattening.

Section 4: Discussions on radius choice of the azimuthal-quadratic phase mask

To obtain fine focusing patterns through PMT, the radius of the phase mask should be optimized. Here we take a left-handed circularly polarized (LCP) vortex beam with a topological charge of l = 10 as an example. By exploiting the vectorial angular spectra theory, the focusing patterns through an azimuthal-quadratic phase mask with the same coefficient l_0 = 40 but different radius are presented in Fig. S3. We can see that when the inner radius is 0 μm (i.e., a common circular shape phase mask) the intensity spreads along the azimuth and forms a sweat-heart shaped pattern. Therefore, the most intensity is not well focused into a spot. By increasing the inner radius to 50 μm, the intensity spreading is gradually suppressed. With a further increment of the inner radius, the intensity spreading phenomenon emerges again. Therefore, for obtaining a fine focusing pattern and improving the intensity utilization, the inner radius is selected as 50 μm when the outer radius is 80 μm. Note that, when the inner radius and outer radius are simultaneously scaled, the focusing pattern just scales with a ratio.

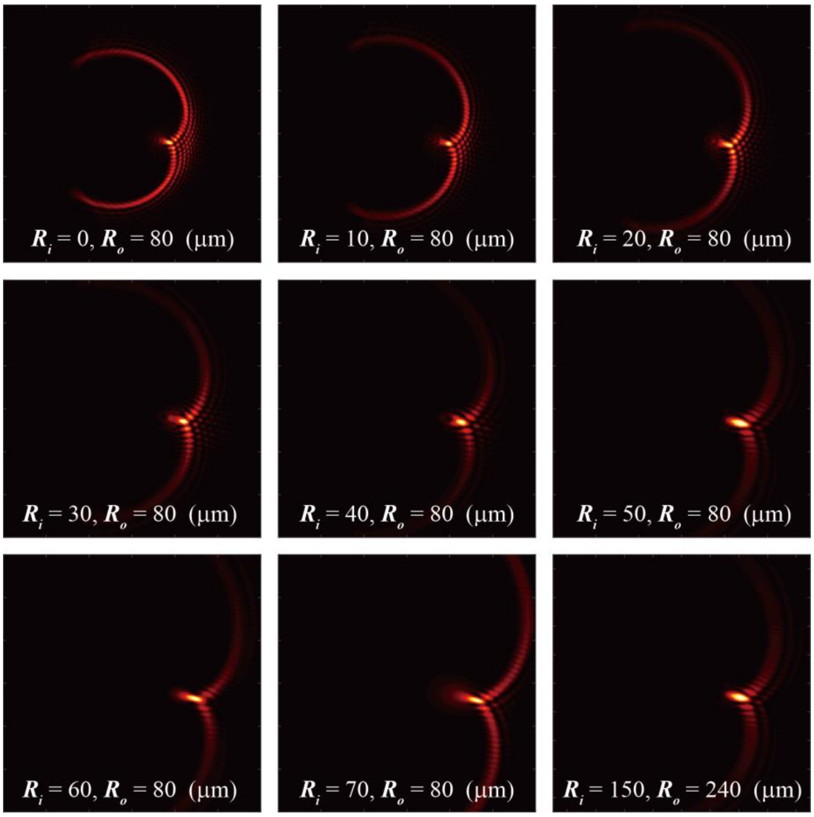

Fig. S3. Focusing patterns through the azimuthal-quadratic phase masks with different radius.

Section 5: Experiment setup and measurement procedure

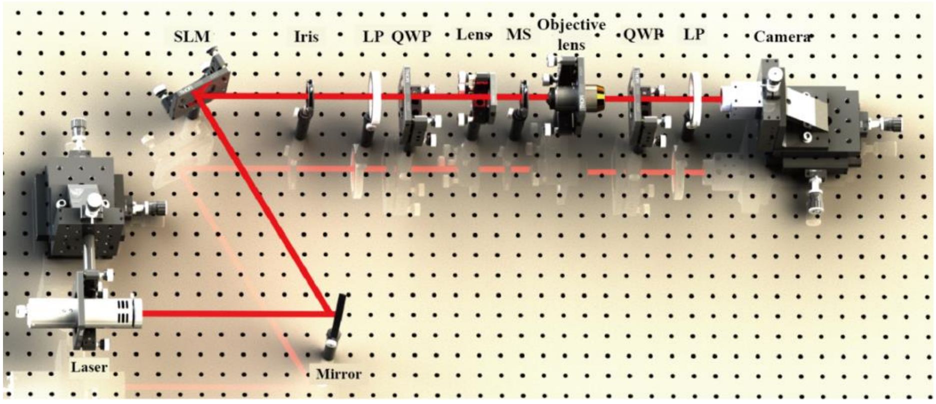

Fig. S4. Experimental setup. Optical elements: spatial light modulator (SLM), linear polarizer (LP), quarter-wave plate (QWP), metasurface (MS).

Measurement procedure:

The light from the He-Ne laser is modulated by a reflective SLM, which can generate anticipated vortex beams via computer generated holograms. We use an iris to pick up only one diffraction order and the combination of a linear polarizer (LP) and a quarter-wave plate (QWP) to generate LCP incidence. The generated LCP OAM is then focused by a lens so that its lateral size matches with the size of the metasurface based phase mask. When light passes through the metasurface, a $50\times$ objective lens mounted on a 2D translation platform is utilized to magnify the optical patterns on the camera. The 2D translation platform is firstly tuned to make the metasurface located at the imaging plane of the objective lens so that we can check whether good alignment between the metasurface and OAM modes generated by SLM is achieved. Subsequently, the imaging plane of the objective lens is changed to the focusing plane of the phase mask and another pair of QWP and LP is inserted before the camera to eliminate the co-polarization components.

Section 6: Diffraction efficiency of the fabricated geometric metasurface

To characterize the diffraction efficiency of the metasurface, a geometric phase metasurface-based beam deflector with a deflection angle of 15° is designed and fabricated. As indicated in Fig. S5(A), although the TiO_2 nanopillars are separated from each other, there are also some fabrication errors, which causes a degraded diffraction efficiency compared with the design. The measured diffraction efficiency around 633 nm is about 30%, as shown in Fig. S5(B).

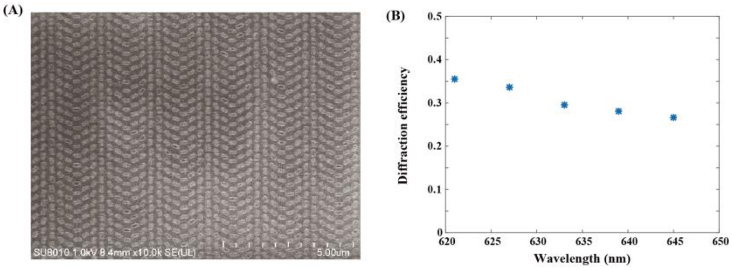

Fig. S5. The diffraction efficiency of the fabricated geometric metasurface. (A) The scanning electron microscope (SEM) of a geometric metasurface-based beam deflector. (B) Measured diffraction efficiency around a wavelength of 633 nm.

Section 7: Detection efficiency of the geometric metasurface

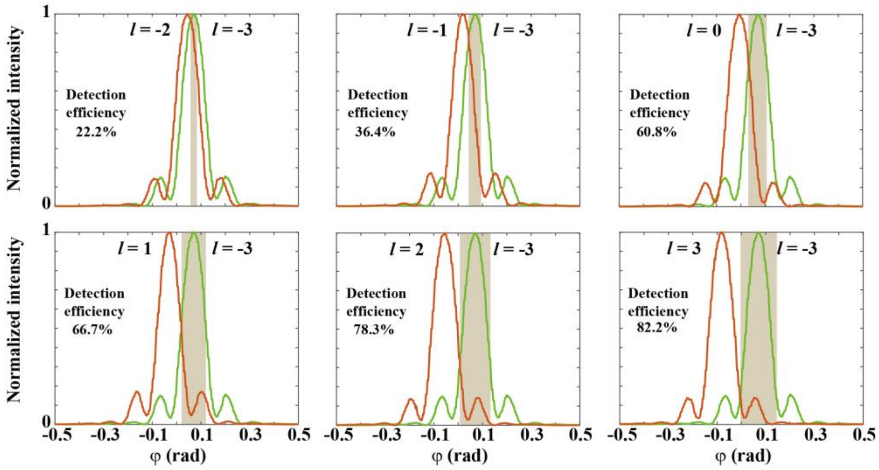

Fig. S6. Detection efficiency of the geometric metasurface. Intensity curves of two different OAM modes with different mode intervals. Detection efficiency is defined by intensity fraction in the weak cross-talk region (indicated by the shadow area).

To quantitatively characterize the extent of mode overlap, we define the detection efficiency as the intensity fraction in the weak cross-talk region, indicated by the shadowed areas in Fig. S6. When Δl = 1, there is about 77.8% intensity overlap

between them and only one peak appears at the combined intensity curve, which cannot be distinguished. With the expansion of mode interval from Δl = 1 to Δl = 6, the detection efficiency increases from 22.2% to 82.2%.

Section 8: Measured errors of azimuthal coordinates in Fig. 2(D)

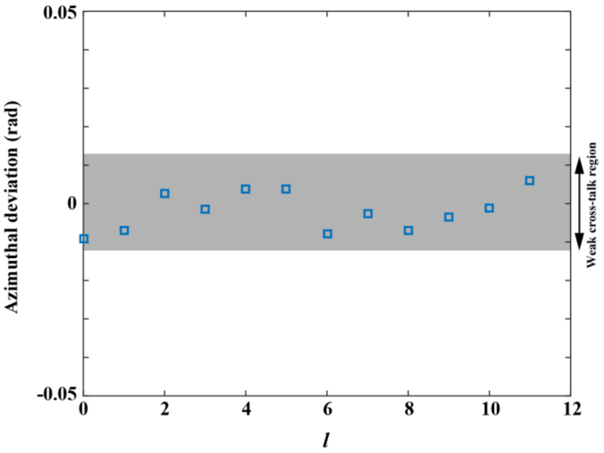

Fig. S7. Measured errors of azimuthal coordinates in Fig. 2(D). The differences between the measured and theoretical azimuthal coordinate are no more than ±0.01 radian. In this situation, the peak intensity is still located within the weak cross-talk area of each mode (For a coefficient l_0 = 40, the azimuthal separation between the intensity peaks of two superimposed adjacent modes is 1/40 = 0.025 radian), as indicated by the shadow area.

Section 9: Discussions on the centre-misalignment in measurements

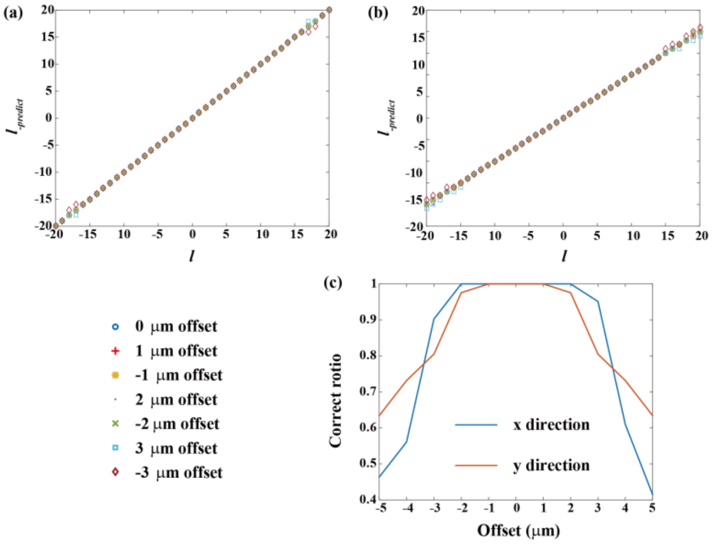

Fig. S8. Influences of centre misalignments between the metasurface and received vortices. (a) (b) Simulated detection results with different position offsets. (a) When the position offset is along the *x* direction. (b) When the position offset is

along the y direction. (c) The correct ratio of detection within ±20 OAM modes with different position offsets. When the centre-misalignment is beyond ± 3 μm, the correct ratio drops below 90%.

Section 10: Simulated results of LCP vortices up to 100 orders at 633 nm

The intensity patterns for arbitrary OAM modes up to 100 orders with an interval of 1 are displayed in Fig. S9~S14. We can see that all the focusing patterns locate on a fixed circle without obvious pattern variation and there is only an azimuthal rotation for different OAM modes.

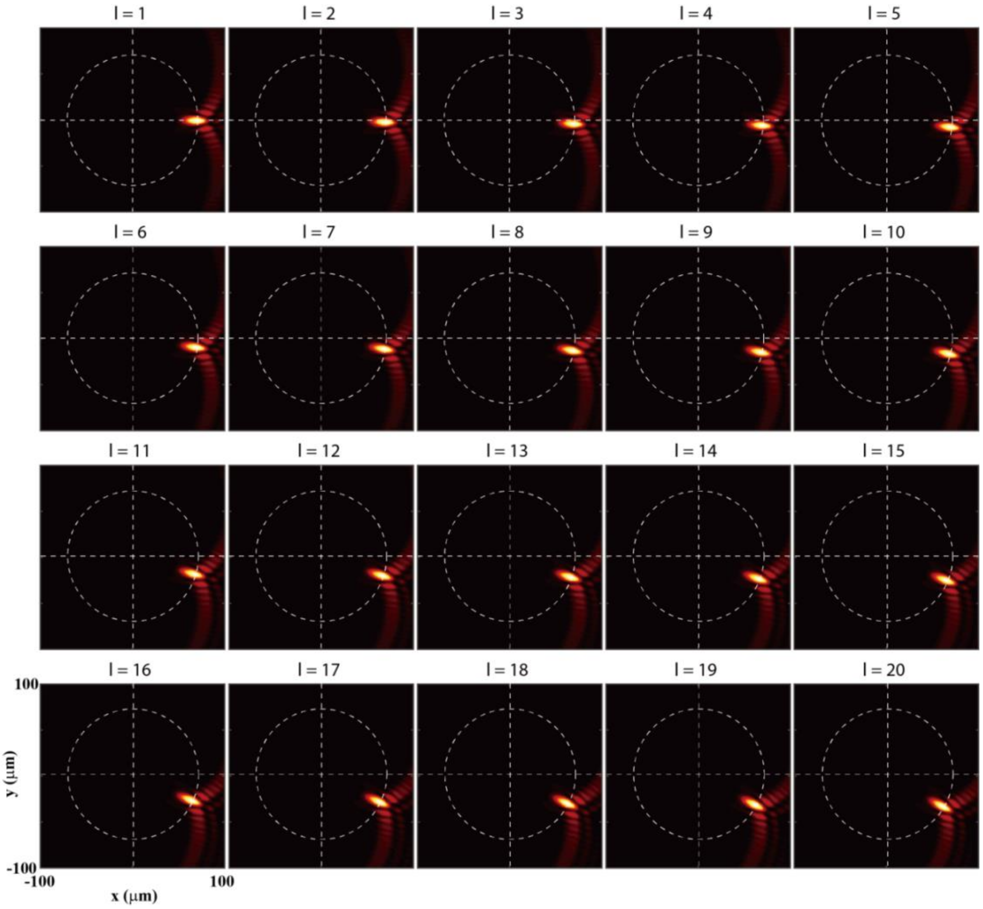

Fig. S9. Simulated focusing patterns for LCP OAM modes from *l* = 1 to *l* = 20.

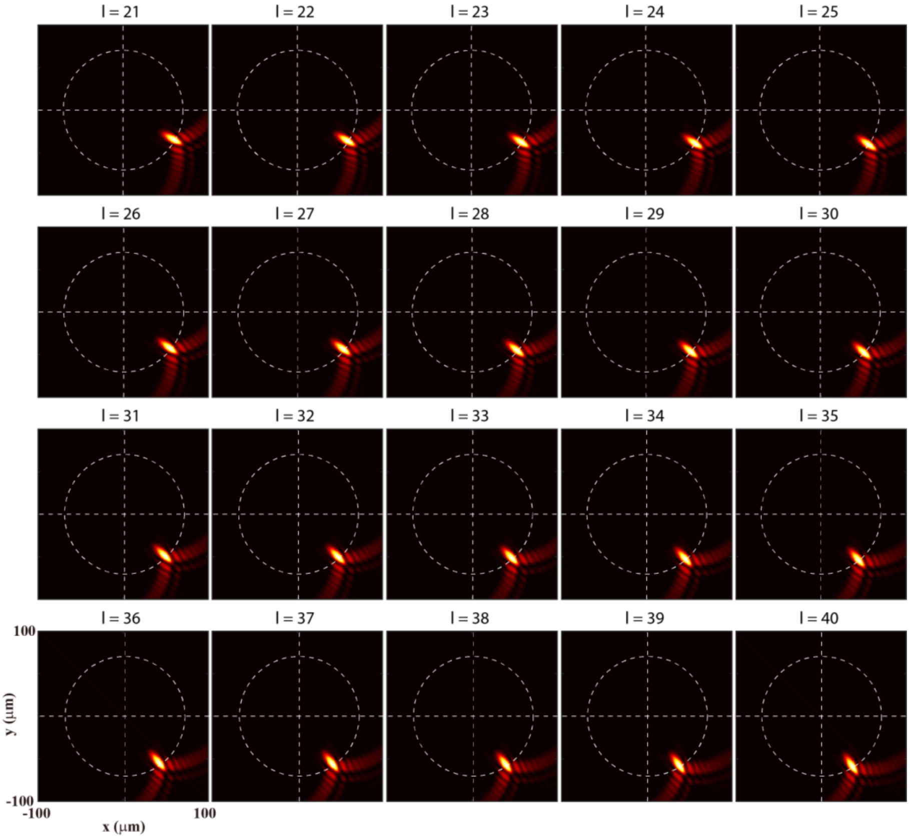

Fig. S10. Simulated focusing patterns for LCP OAM modes from *l* = 21 to *l* = 40.

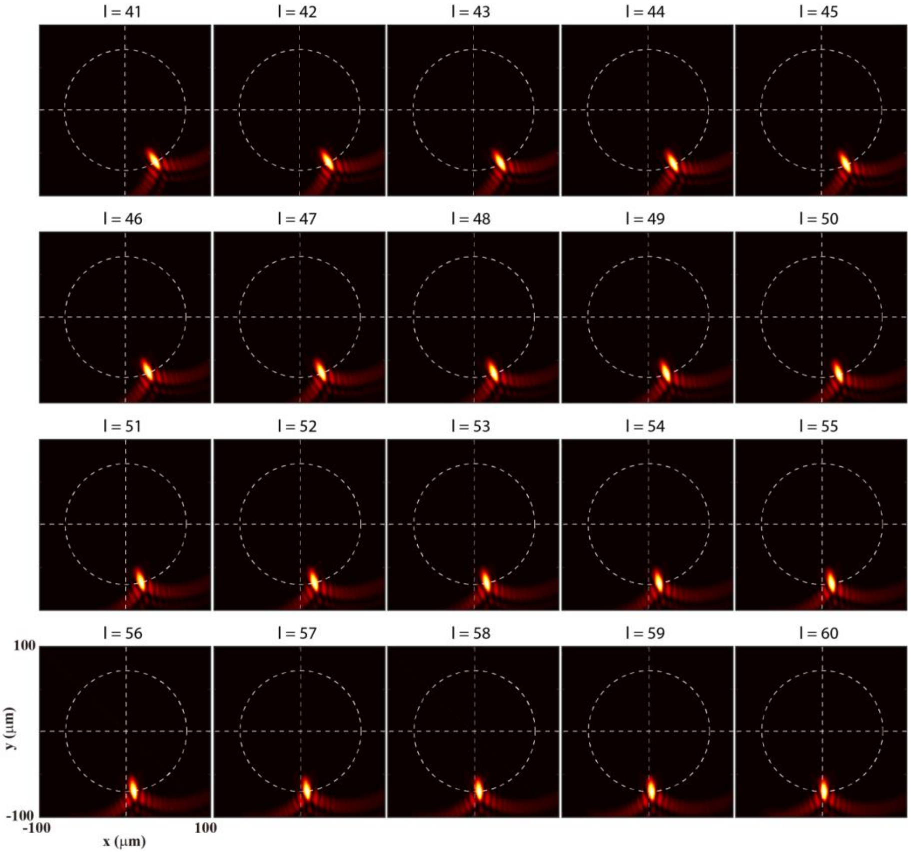

Fig. S11. Simulated focusing patterns for LCP OAM modes from *l* = 41 to *l* = 60.

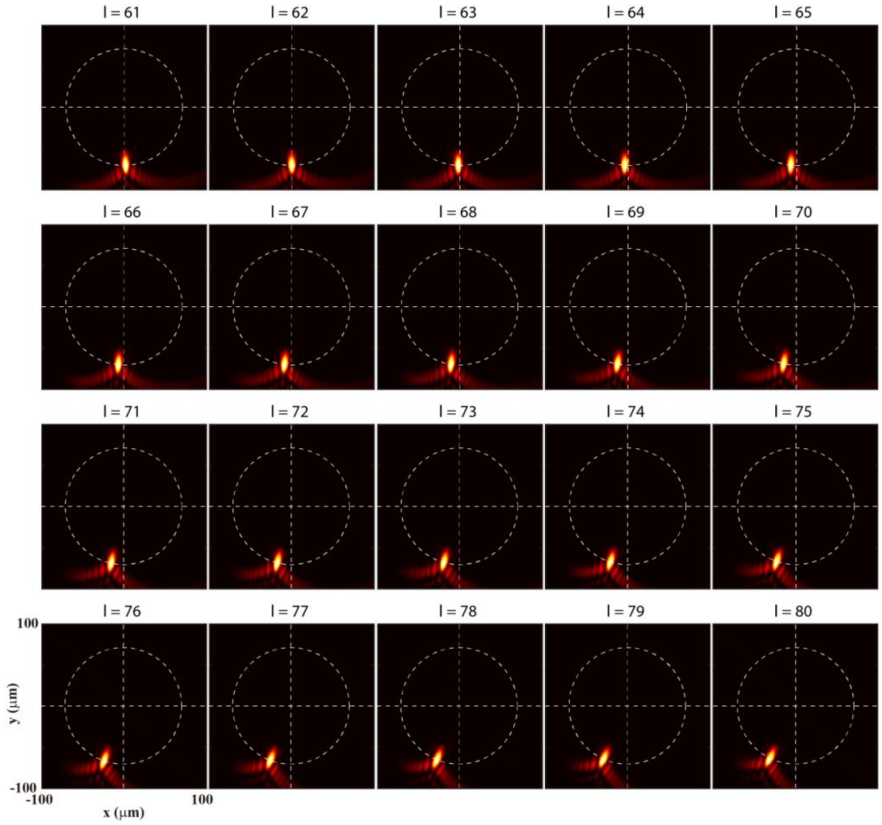

Fig. S12. Simulated focusing patterns for LCP OAM modes from *l* = 61 to *l* = 80.

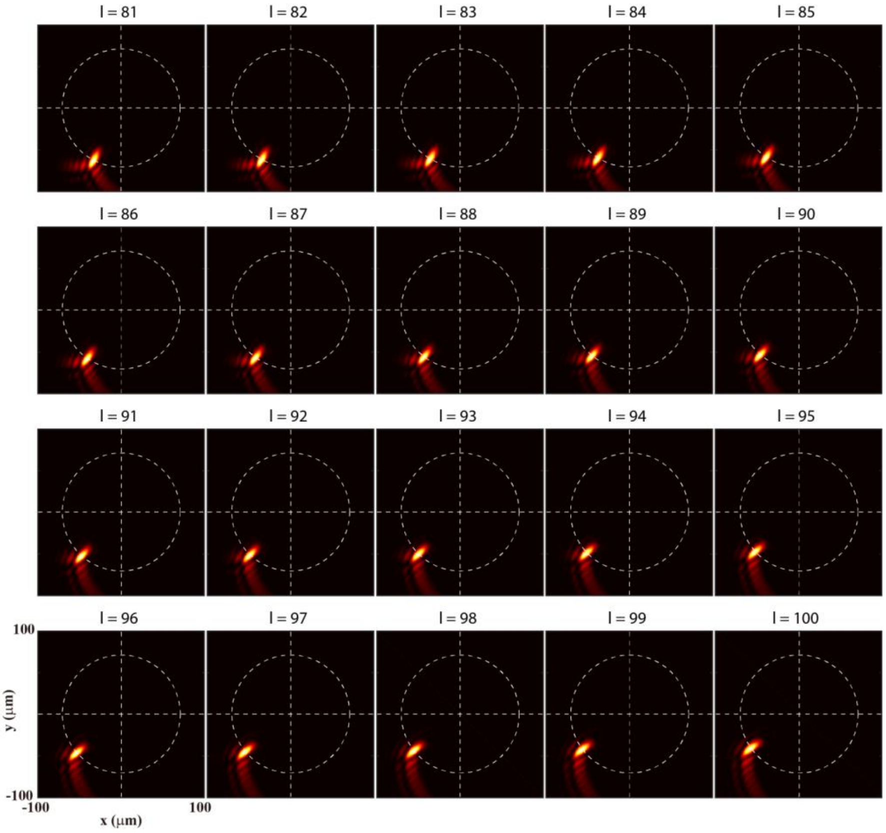

Fig. S13. Simulated focusing patterns for LCP OAM modes from *l* = 81 to *l* = 100.

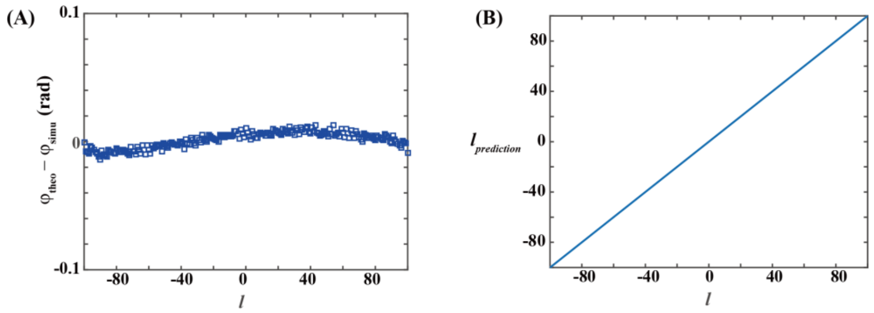

Fig. S14. Numerical errors and detection results. (A) Numerical errors of azimuthal coordinates for LCP OAM modes from l = -100 to l = 100. (B) Simulated detection results within ±100 orders

Section 11: Simulated polarization conversion ratio (PCR) of the spin-decoupled metasurface

Since the eight nanostructures including four fundamental nanoposts with different length and width (L, W) and their orthogonal structures with length and width (W, L), only the first four nanostructures are simulated, with their PCR shown in Fig. S15.

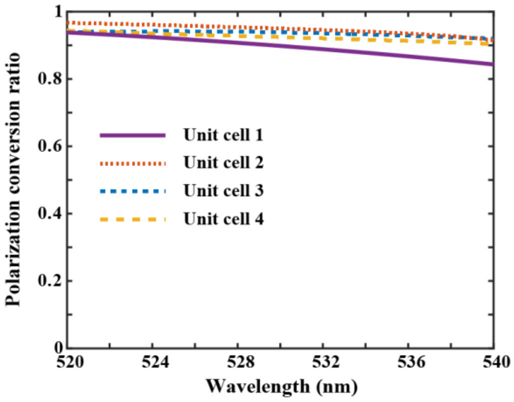

Fig. S15. Simulated PCR of the spin-decoupled metasurface. .

Section 12: Experimental demonstrations of linearly polarized (LP) vortex sorting at 532 nm

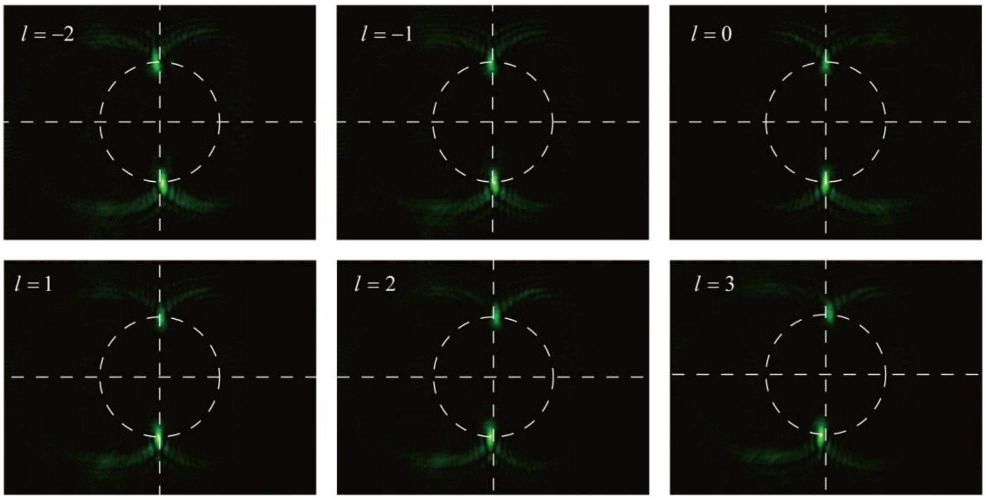

Fig. S16. Experimental demonstrations of linearly polarized vortex sorting at a wavelength of 532 nm. Measured focusing patterns for different OAMs. For linearly polarized vortices that can be taken as superimposed of LCP vortices and RCP vortices with equal weight, two focusing patterns are locating at two centre-symmetric positions on the white dotted circle through spin-decoupled PMTs. In the measurement procedure, the two half-wave plates before and behind the metasurface are removed. The slightly un-equal intensity of the two focusing patterns may be attributed to the imperfect metasurface fabrication and measurement.

Section 13: Sorting of superimposed OAM modes

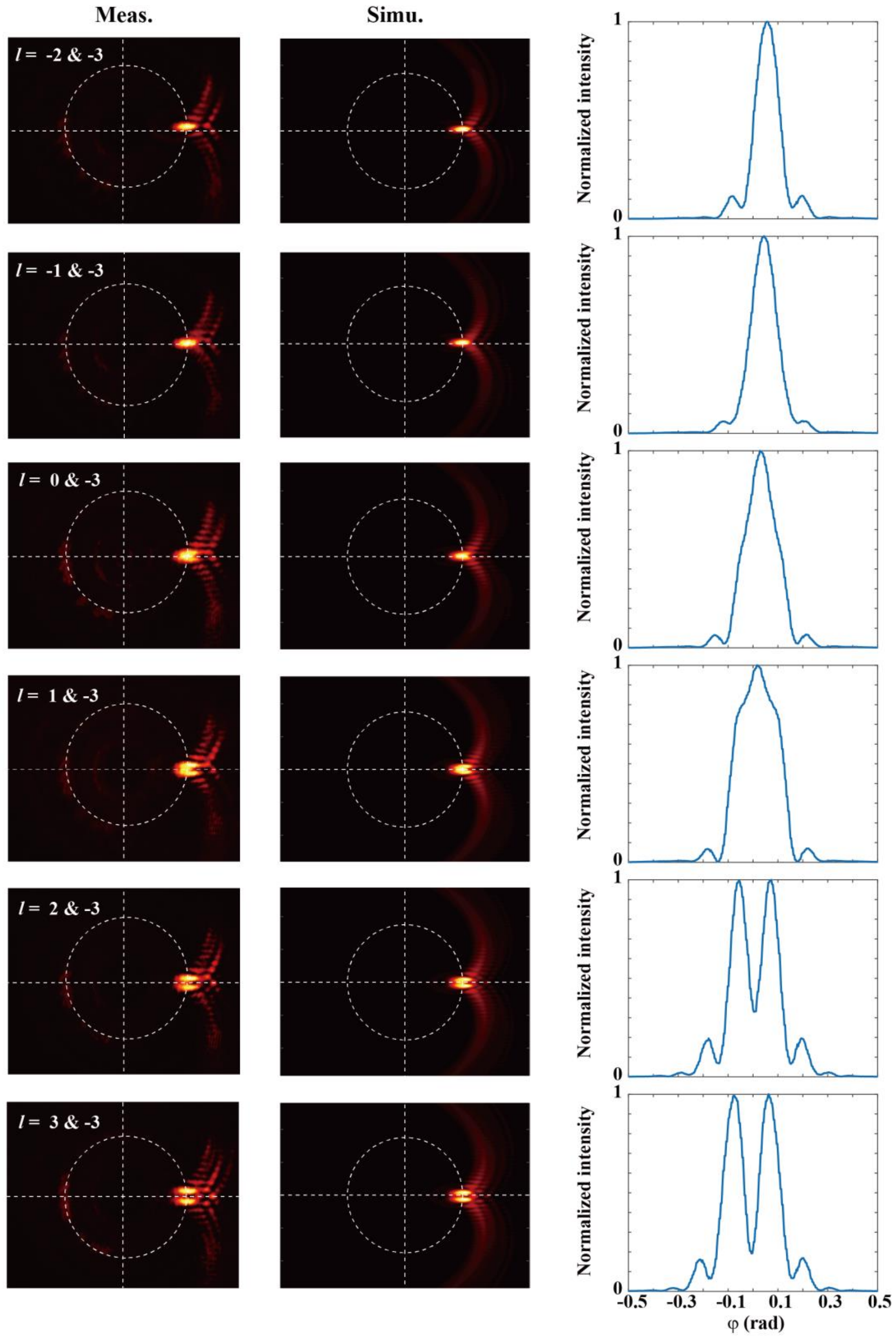

Fig. S17. Focusing patterns of two superimposed OAM modes with different mode intervals at a wavelength of 633 nm. Left panel: measured intensity patterns. Middle panel: simulated intensity patterns the combined intensity curves of two superimposed OAM modes with different mode intervals. Right panel: intensity curve along the white dotted line in middle panel. The mode interval changes from Δl = 1 (uppermost) to Δl = 6 (lowermost).

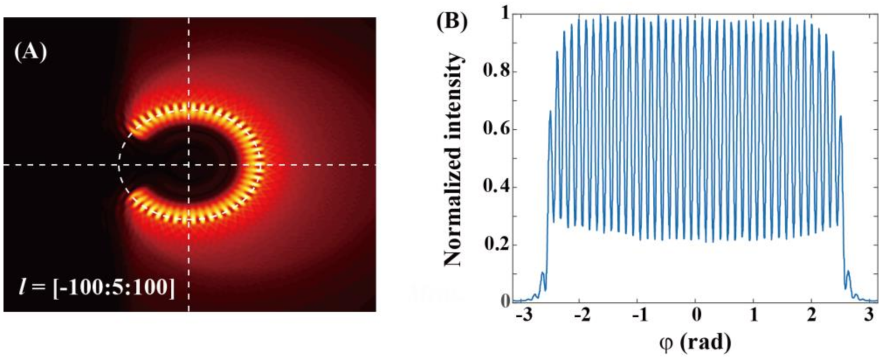

Fig. S18. Simulated results of PMTs with 41 superimposed OAM modes with a mode interval of Δ*l* = 5. (A) Simulated focusing patterns. (B) Intensity curves along the white dotted circle line in (A).

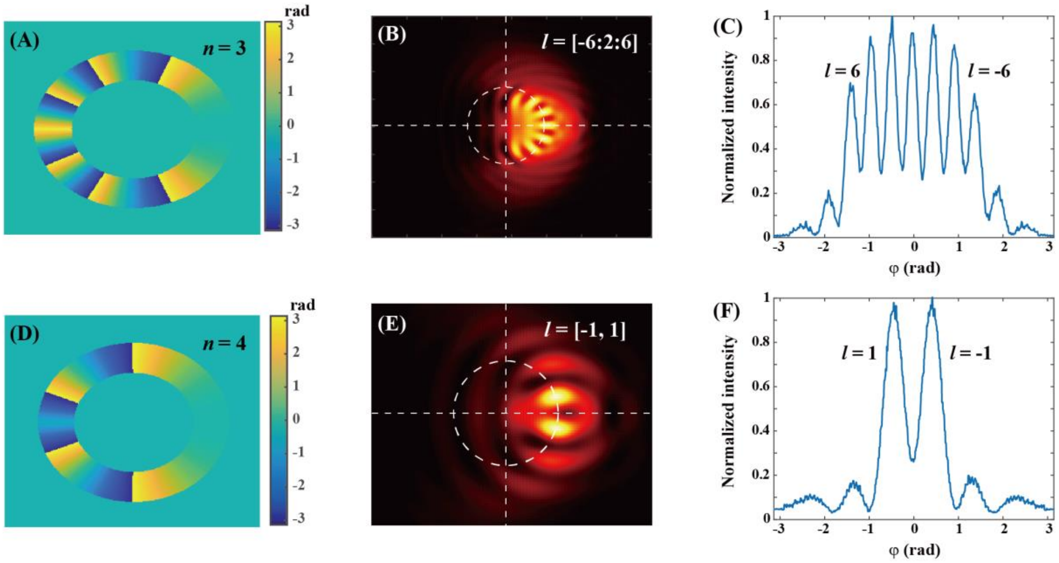

Fig. S19. Simulated results of PMTs via modified phase masks. (A) and (D) The phase profiles of modified phase masks. (B) and (E) Simulated focusing patterns of superimposed vortices. (C) and (F) Intensity curves along the white dotted circle line in (B) and (E). (A)~(C) n = 3 and (D)~(F) n = 4.

Note that, the equivalent focal distance of the modified phase mask can be deduced as:

$$f' = \frac{n^2 k_0 r_0^2}{l_0} \tag{10}$$

which increases sharply with n. As a consequence, the effective numerical aperture of the phase mask decreases and the full width at half maximum of focusing pattern obviously broadens, as indicated in Fig. S19(D~F), which impedes the further

improvement of the mode resolving power. Besides, the much slow azimuthal phase change makes the equivalent wavevector in the whole azimuthal coordinates become propagation wave. The interchange effect between the propagation wave region and evanescent wave region under the excitation of vortices, as illustrated in the phenomenological explanation model of PMT (Section 2 of the Supplementary file), cannot be maintained again.

Section 14: Broadband PMTs and broadband vortex sorting

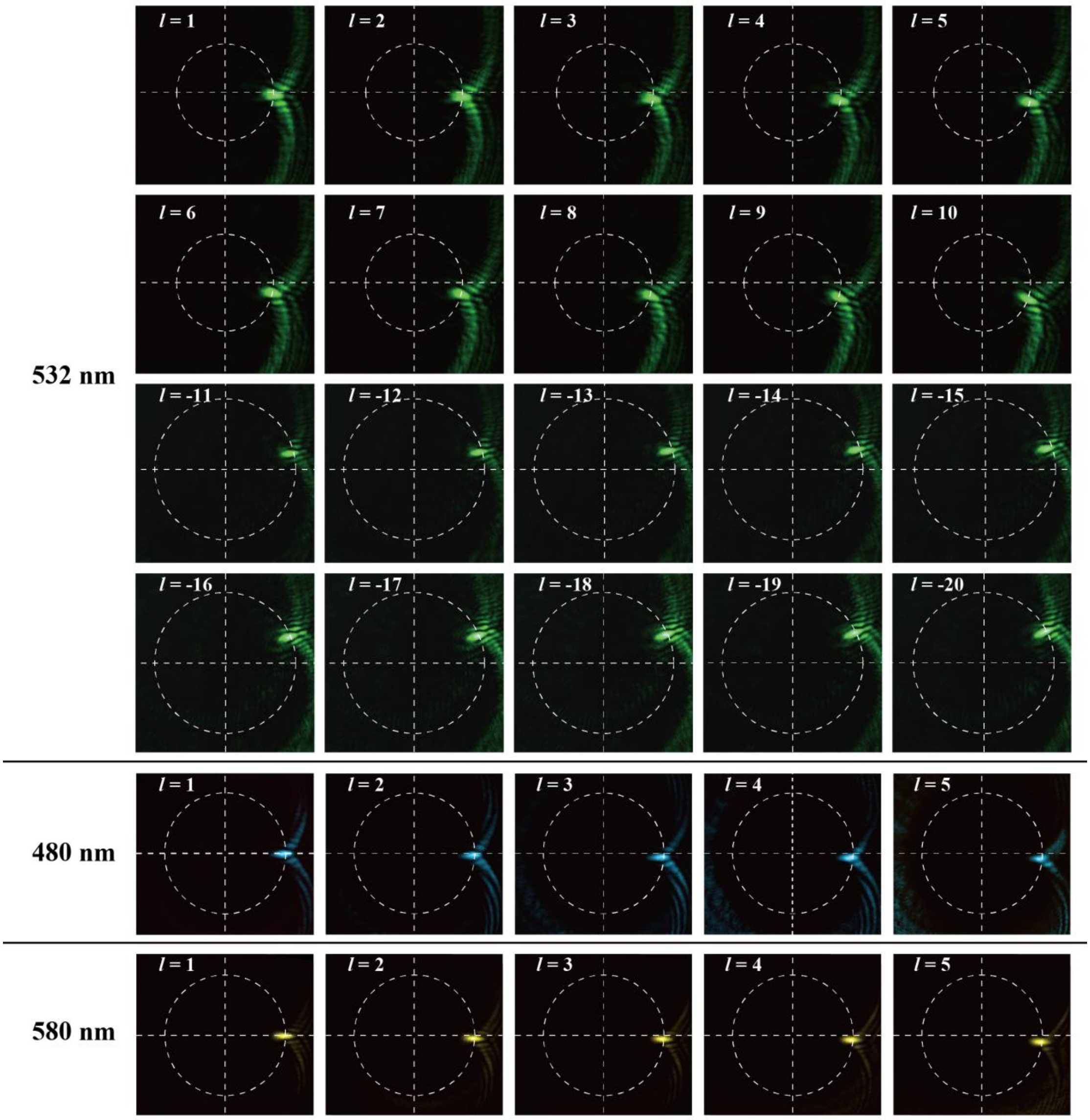

Fig. S20. Experimental demonstration of LCP OAMs sorting at wavelengths of 532, 480, and 580 nm.

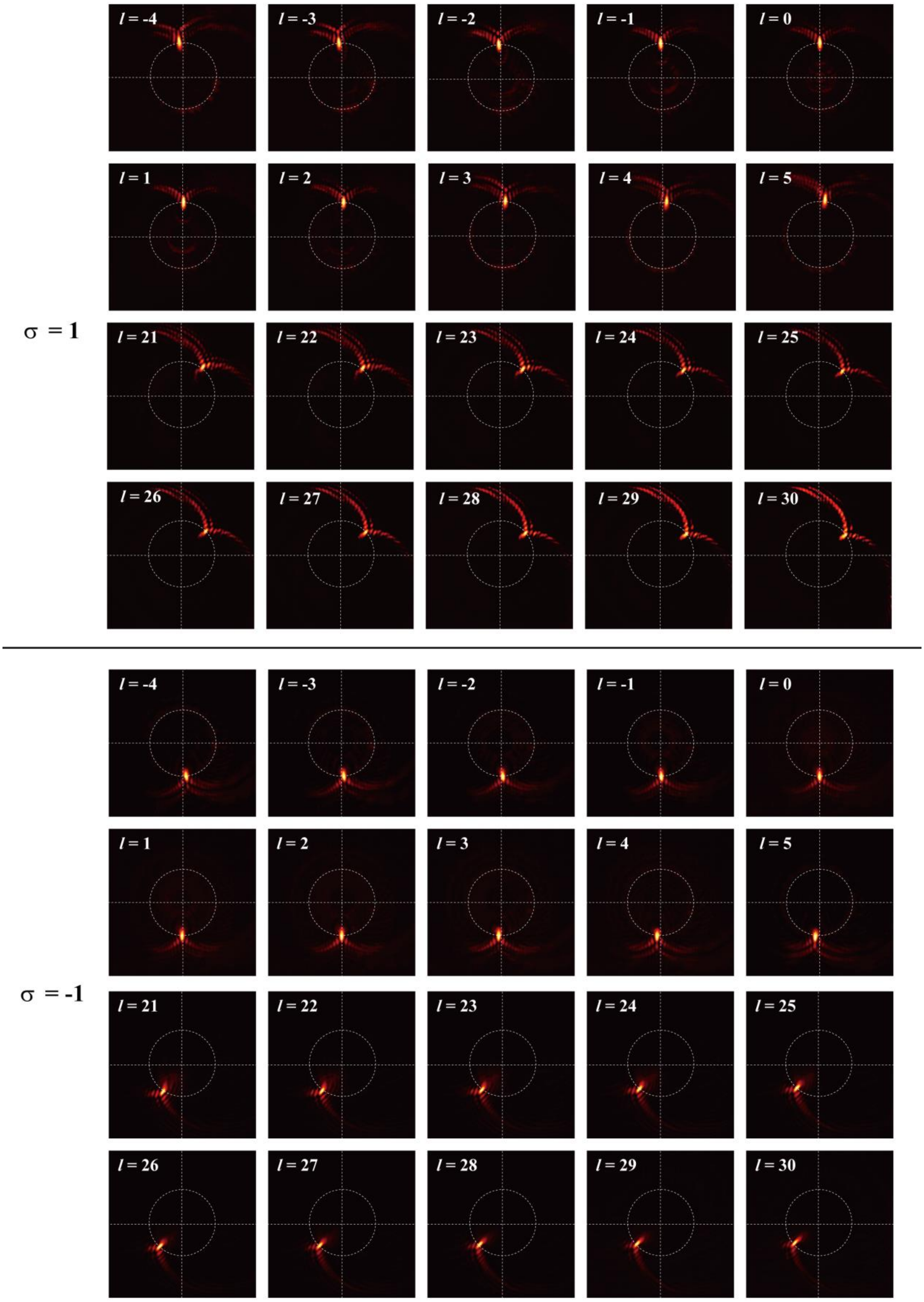

Fig. S21. SAMs and OAMs sorting at a wavelength of 633 nm. Measured focusing patterns for different SAMs and OAMs with the topological charge changing from -4 to 5 and from 21 to 30.

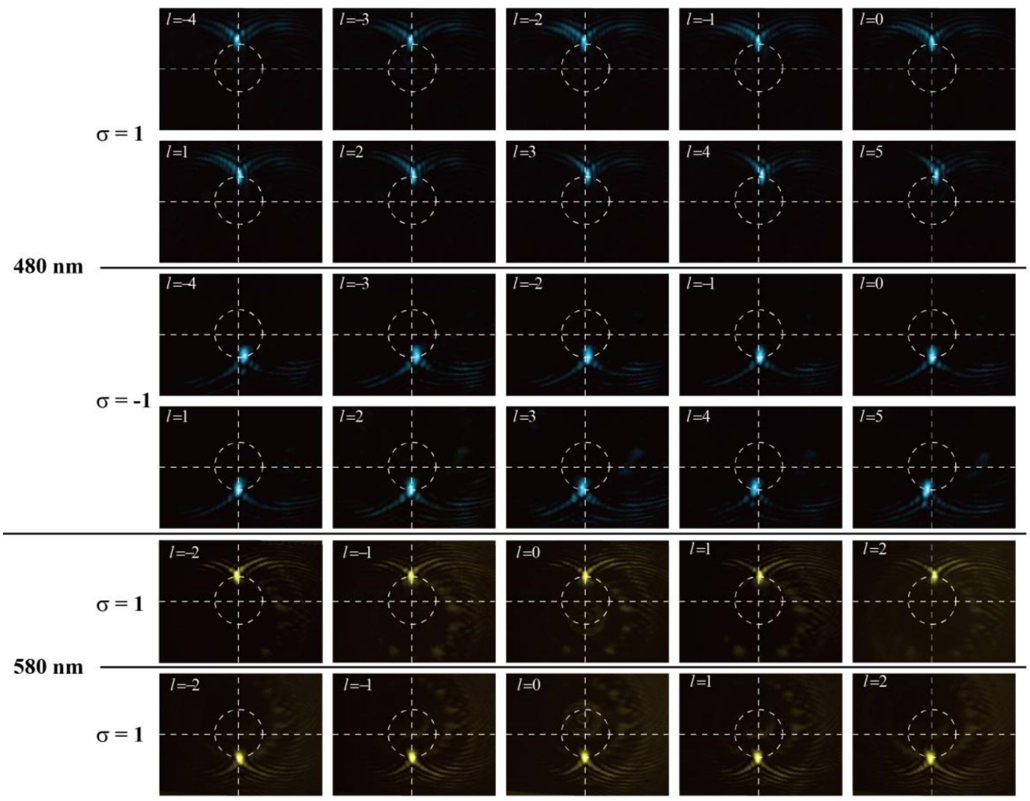

Fig. S22. SAMs and OAMs sorting at wavelengths of 480 and 580 nm. Measured focusing patterns for different SAMs and OAMs.

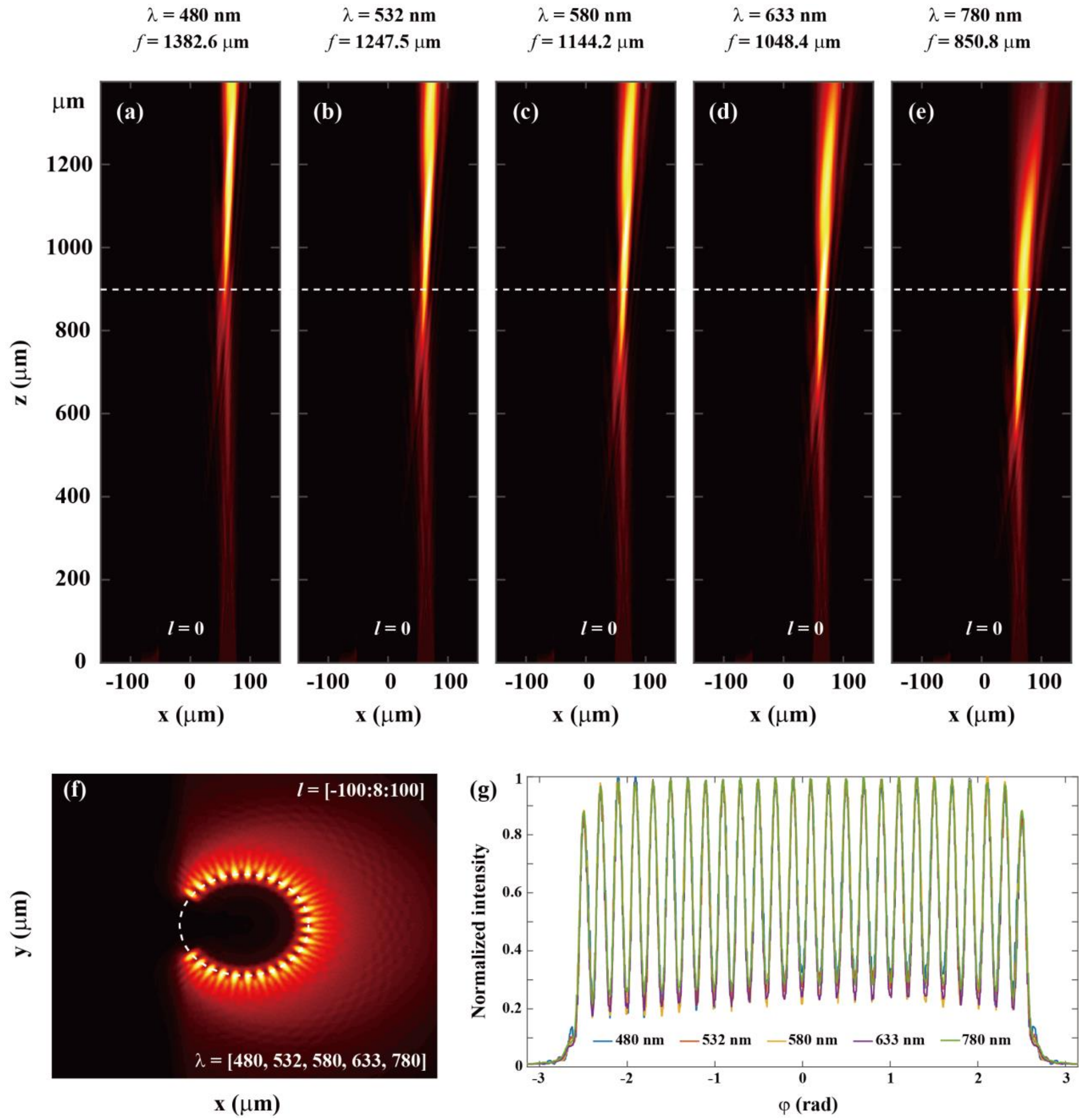

Fig. S23. OAMs sorting of broadband LCP vortices. Simulated focusing patterns of a plane wave in the *xoz* plane at different wavelengths of (a) 480, (b) 532, (c) 580, (d) 633, and (e) 780 nm. (f) Simulated combined focusing patterns of superimposed vortices at different wavelengths on a transverse plane, indicated by the white dotted line in (a)~(e). (g) Intensity curves along the white dotted line in (f).
